# Three-year-cohort-study: clinical and cost effectiveness of an inpatient psychiatric rehabilitation

**DOI:** 10.3389/fpsyt.2023.1140265

**Published:** 2023-04-28

**Authors:** Mahmoud A. Awara, Laura M. Downing, Dorothy Edem, Norma Lewis, Joshua T. Green

**Affiliations:** ^1^Department of Psychiatry, Dalhousie University, Medical School, Halifax, NS, Canada; ^2^The College of Physicians and Surgeons of Nova Scotia, Halifax, NS, Canada; ^3^The Royal College of Psychiatrists, London, United Kingdom; ^4^Mental Health and Addictions Services, Nova Scotia Health Authority, Halifax, NS, Canada

**Keywords:** inpatient psychiatric rehabilitation, clinical effectiveness, quality of inpatient psychiatry care, cost effectiveness, the effectiveness of inpatient psychiatry rehabilitation for severe and persistent mental illness

## Abstract

**Introduction:**

There has been a resurgence of interest in psychiatric rehabilitation to cater to patients with chronic and complex mental illnesses.

**Aims:**

This study is aimed at examining patients' characteristics and the prevalence of psychiatric and non-psychiatric comorbidity in a local inpatient rehabilitation service, as well as to investigate the impact of the whole-system approach to rehabilitation on future utilization of mental health services and to analyze the cost-effectiveness and quality of this service.

**Method:**

Patients managed over 3 years in a psychiatric rehabilitation inpatient unit were self-controlled; they were retrospectively (pre-rehabilitation) and prospectively (post-rehabilitation) examined for readmission rate, length of stay (LOS), and emergency room (ER) visits. Relevant information was retrieved from Discharge Abstract Database (DAD), Patient Registration System (STAR), and Emergency Department Information System (EDIS). The quality of care in the rehabilitation unit was examined using the Quality Indicator for Rehabilitative Care (QuIRC), and the cost analysis was conducted using data obtained from a single-payer government medical service insurance (MSI) billing system.

**Results:**

Of the 185 patients admitted over the study period, 158 were discharged. There was a significant reduction in readmission rate (64% decrease), LOS (6,585 fewer days spent in hospital), and ER presentations (166 fewer visits) (*P* < 0.0001), respectively. There were substantial subsequent cost savings in the post-rehabilitation year.

**Conclusion and implications for practice:**

In the 3-year study, an inpatient psychiatric rehabilitation service in Nova Scotia, Canada, resulted in the successful discharge of most patients with severe and persistent mental illness to more socially inclusive environments. It also reduced their post-rehabilitation mental health service utilization, hence greatly enhancing the effectiveness and efficiency of these services.

## Introduction

For more than 15 years, there has been a renewal of interest in psychiatric rehabilitation using whole-system approaches at national and international levels (1). This resurgence of interest has arisen in pursuit of improving the life experience of people with chronic and complex mental health conditions.

Psychiatric rehabilitation uniquely emphasizes the “bio-psycho-social” model and the concepts of recovery as an outcome for patients with endured and complex mental health challenges (2). A wide variety of definitions have been subsumed under the rubric of rehabilitative recovery as a value-led practice, which includes the following: (a) clinical recovery that is defined as recovery from symptoms and difficulties in response to effective care and treatment; (b) personal recovery, which is recovery with an active commitment to achieving a cherished pattern of life and living with or without symptoms; (c) recovery-oriented approaches, whereby patient-centered and goal-directed approaches are implemented by staff qualified and skillful in the recovery and integration process; and (d) the recovery movement, which involves multidisciplinary professionals and stakeholders collaborating and unifying endeavors to develop and transform mental healthcare and treatment (3).

In the current era of clinical practice and increased economic pressures, there is no place for complacency in mental healthcare; hence, services must provide ongoing evidence to assure quality and justify the continued investment in said services. Various stakeholders including patients, families, care providers, administrators, policymakers, and the government should see that the money spent on mental health services is cost-effective and delivers good outcomes for service users (2).

However, the measurement of outcomes in mental health services is contentious and sometimes difficult to define. In this area, researchers may face different perspectives on what outcomes are desired and what constitutes high-quality care. Quality is also a stakeholder-relative concept and may be construed differently by service users, families, clinicians, and the government. Outcome measures of psychiatric rehabilitation services become even more intricate and compounded by the multimodal complexity of mental health rehabilitation services. In addition, economic austerity and a lack of resources could oscillate inadvertently between the outcomes to be measured, which could represent another challenge that researchers in this field have yet to overcome.

A recent systematic review of the effectiveness of mental health rehabilitation services has demonstrated a consistent reduction of inpatient service use after an inpatient rehabilitation admission and/or successful discharge to a supported accommodation service compared to the period before the admission (4).

The National Institute for Health and Care Excellence (NICE) has explicitly incorporated psychiatry rehabilitation in its recent clinical guideline (NG181), 2020 as an important treatment for patients with complex psychosis that should be embedded in a local comprehensive mental health service. The rehabilitation treatment should be recovery-oriented, patient-centered, and be offered in the least restrictive environment with the recognition that some patients may require a long-term supported placement post-rehabilitation.

The cost-effectiveness argument has been a very important part of making the case for ongoing investment in mental health rehabilitative services (5). In the UK, expensive out-of-area treatments (OATs) for patients with severe and complex mental health needs have led to renewed interest in investing in psychiatric rehabilitation services.

Making a case for investment in psychiatric rehabilitation services must be based on evidence of their quality of care and clinical effectiveness (2). This may include reducing the length of stay (LOS), hospital readmission rate, and other psychiatric services' utilization, e.g., short-stay unit (SSU) and emergency room (ER) visits. It should also be demonstrated that rehabilitation patients can be discharged to a more independent place of residence and acquire employability skills (6).

The objectives of our study were, therefore, to (i) examine the demographics of a cohort of patients receiving recovery-oriented psychosocial psychiatric rehabilitation (e.g., age, gender), (ii) assess the rate of psychiatric and non-psychiatric comorbidity in this cohort, (iii) investigate the impact of a psychiatric rehabilitation model (whole-system approach) on LOS, readmission rate, and ER visits, (iv) examine the quality of care of service provision using the Quality Indicator for Rehabilitative Care (QuIRC), (7–11), and (v) examine the cost-effectiveness/reduction of inpatient rehabilitation.

## Methods

### Study design

The study retrospectively examined inclusively all the patients who had been discharged within 3 years and 1 month period between 1 June 2012 and 30 June 2015.

The study hypothesizes that inpatient psychiatric rehabilitation is clinically effective and that the whole-system approach of the biopsychosocial rehabilitation model has a carryover therapeutic effect. This effect can be measured by comparing service utilization in the pre- and post-rehabilitation treatment in patients with severe and persistent mental illness.

Each patient's service utilization of the acute psychiatric services (rate of admissions and LOS) and ER visits were examined for a 1-year period in a bi-directional manner for the pre- and post-exposure to the rehabilitation treatment in the unit.

Information was retrieved from the Discharge Abstract Database (DAD) to examine the outcome of patients who were admitted and discharged over the study period. The service utilization information for each discharge was retrieved from the STAR practice management system in this context.

Additional variables, such as the primary diagnosis for inpatient rehabilitation admission, other psychiatric and non-psychiatric (**Table 5**) diagnoses, and information on the involuntary/voluntary nature of the admissions were obtained from the DAD. The ER visit data were obtained from the Emergency Department Information System (EDIS) for the same timeframe, and the presentations with mental health problems or diagnoses upon ER discharge were examined.

The estimated cost analysis was conducted using financial data obtained from the Financial Services Office of the local health authority. The costs were based on the Medical Service Insurance (MSI) billing, which amounted to CAD 1,400 per day spent in acute mental health services and CAD 309 per ER visit.

Statistical analysis was conducted with a 95% confidence interval (α = 0.05) using SAS JMP version 12.0. Matched paired *t*-tests were conducted to compare the rate of admission and the total LOS in the pre- and post-rehabilitation treatment period. In addition, descriptive statistics such as mean, median, standard deviations, and 95% confidence intervals were also computed.

The Quality Indicator for Rehabilitative Care (QuIRC) was employed to examine the quality of care in the unit. QuIRC is a standardized, international quality assessment tool for inpatient and community rehabilitation units and although completed by managers of the service, during its development, the ratings were validated against service users' experiences of care and autonomy and found to correlate well (9).

The QuIRC tool assesses the provision of care across seven domains: (1) living environment, (2) therapeutic environment, (3) treatments and interventions, (4) self-management and autonomy, (5) social interface, (6) human rights, and (7) recovery-based practice. The tool is the product of collaborative work involving 11 centers in 10 European countries and was determined based on the evidence on crucial components of care gleaned from different studies and systematic reviews (7–11).

### Overview of the setting

The study was conducted on a 40-bed inpatient mental health rehabilitation unit in Dartmouth, Nova Scotia, Canada, serving a population of ~357,000 (12). During the study period, the rehabilitation unit received 185 admissions.

The studied unit is an open community psychosocial rehabilitation service that provides a recovery-focused approach to adult patients with severe and persistent mental illness. The unit is one of the two 24/7 inpatient rehabilitation services (a second is a locked unit) providing psychiatry rehabilitation services for the population of Nova Scotia in Canada. The unit is operating under the auspices of the Mental Health and Addictions Program (MHAP)/Recovery and Integration Services (R&I) which is part of the Provincial Health Care System in Nova Scotia, Canada (Nova Scotia Health Authority).

The unit is based in the community and was opened in 2012 with 40-beds capacity. The unit has no maximum length of stay, and the bed capacity was later reduced to 20 beds with a 75% occupancy.

The unit has 45 full-time equivalent (FTE) staff, which includes one FTE psychiatrist, two psychiatric residents (~0.8 FTE each), one family physician, two FTE occupational therapists, 32 FTE nurses, five FTE unlicensed support workers, one FTE social worker, one FTE recreation therapist, and one FTE volunteer. Peer support workers with past mental illness experience are also employed as staff to support patients. The aforementioned staffing level was based on 40-beds capacity.

The unit is diversified and has extended referral criteria and a recovery-oriented philosophy (4, 13); it accepts general adult patients from different destinations, including (a) slow-to-remit patients from acute services who are likely to require intensive support following discharge, (b) long-stay patients who are functionally not ready for community placement because of unremitting symptoms and/or deficits skills, (c) patients with acute exacerbation on top of enduring and deteriorating mental illnesses that impact on their global level of functioning, and (d) patients who need the alternate level of care (ALC) and would have been discharged if highly supported accommodation had been available in the community.

### Biopsychosocial treatment approach in the rehabilitation unit

Each patient admitted to the rehabilitation unit underwent a detailed assessment process involving an interprofessional team and included the patient, family, nurses, social workers, occupational therapy, recreation therapy, and psychiatrists, to collaboratively assess the nature and degree of their illness and to develop a comprehensive biopsychosocial management plan (2). The biological treatment was aimed at ameliorating symptoms, reducing distress, and speeding readiness for the rehabilitation process using the best available evidence and guidelines (14–16).

Simultaneously, patients' psychosocial rehabilitation goals were determined and worked upon with support from comprehensive occupational, psychological, recreational, and social assessments that consider current strengths and areas requiring treatment and skill development. Using a shared decision-making framework, recovery goals were defined with involvement from patients and families and were aimed to achieve recovery and integration into the community.

## Results

During the study period, the unit received 185 admissions; there were 14 patients discharged to the community after the timeframe of the study, seven patients were transferred back to the acute services, and six patients remained in the rehabilitation unit waiting for a supported placement in the community. Hence, the 158 patients who were discharged within the timeframe of the study were subsequently examined for outcomes.

Most patients were male (66%) subjects with a mean age of 38. The main diagnosis was schizophrenia which affected 84% of patients. One or two additional psychiatric and/or medical diagnoses, other than the primary diagnosis, were present in 45% of cases. The mean LOS was 165 days, median of 93.5, IQR (interquartile range) 160, and 31% of patients were involuntarily admitted under the Involuntary Psychiatric Treatment Act (IPTA) (Tables 1, 2).

**Table 1:** Cohort characteristics.

**Characteristics**	**Cohort *n* = 158 discharges**
**Primary diagnosis on discharge**	**% of** ***n***
Schizophrenia	84%
Substance use/abuse	4%
Bipolar	4%
Adjustment disorder	2%
Personality disorder	2%
Depression	2%
Other behavioral/emotional disorder ICD-10 (F90-F98)	3%
**Comorbid psychiatric diagnoses on discharge**	**% of** ***n***
None	55%
1 extra	30%
2 or more	15%
**Comorbid medical diagnoses on discharge**	**% of** ***n***
None	55%
1 extra	25%
2 or more	20%
^*^ **Comorbid non-psychiatric conditions/factors on Rehabilitation discharge**	**% of** ***n***
None	8%
1 extra	73%
2 or more	19%
**Involuntary admission**	**% of** ***n***
Yes	31%
No	69%
**Average length of stay in days** **±SD**	**165.7** **±151**
**Gender**	**% of** ***n***
Male	66%
Female	34%
**Average age** **±** **SD (overall and by gender)**	**40.8** **±** **14.8**
Male	38.0 ± 14.0
Female	46.5 ± 14.9

^*^Additional information in **Table 5**.

**Table 2:** Length of stay in the rehabilitation unit as per the primary diagnosis.

**Primary diagnosis**	***N* discharges**	**LOS on the rehab unit (days)**			
		**Median**	**IQR**	**Min**	**Max**
Schizophrenia	133	98	182.5	1	896
Substance use disorder	7	48	21	10	57
Bipolar affective disorder	6	48	200.5	4	248
Personality disorder	3	112	112	59	171
Adjustment disorder	3	57	79	23	102
Depression	3	66	104	1	105
Other behavioral/ emotional disorder	3	105	311	78	389
**All cases**	**158**	**93.5**	**160**	**1**	**896**

IQR, Inter Quartile Range.

The median of the LOS on the rehabilitation unit was higher for patients with personality disorders, behavioral/emotional disorders, and schizophrenia (Table 2).

The mean difference between pre- and post-rehabilitation treatment through the rate of admission, LOS, and ER visits had shown that patients with a schizophrenia diagnosis had significantly lower admission rates, spent fewer days on acute services, and visited the ER less often during the post-rehabilitation year, with a mean difference of 0.86, 41.81, and −1.26, respectively, (*p* < 0.0001). Patients with diagnoses of depression or substance misuse showed a significant reduction in their admission rates and LOS on the acute service but no change in their ER visits (Table 3).

**Table 3:** Summary of the outcomes examining the length of stay, admission rate, and ER visits against different variables.

**Baseline characteristics**	**Total discharges (*N*)**	**1 year pre-rehabilitation**				**1 year post-rehabilitation**						
		**Acute MH Inpatient admissions**			**Total ER visits**	**Acute MH Inpatient admissions**			**Total ER visits**	**Mean difference (post-pre)**		
		**%** ***N*** **discharges (row %)**	**Total admissions Pre**	**Total patient days**		**%** ***N*** **discharges (row %)**	**Total admissions Post**	**Total patient days**		**Acute admissions**	**Acute patient days**	**ER visits**
**Primary diagnosis**												
Adjustment disorder	3	100%	4	206	6	33%	1	6	6	1	66.67	0
Bipolar	6	83%	6	260	9	50%	11	266	22	−0.833	−1	2.167
Depression	3	100%	5	304	2	0%	0	0	2	1.667^*^	101.33^*^	0
Substance use/abuse	7	100%	11	416	23	29%	2	41	18	1.285^**^	53.571^*^	−0.714
Other behavioral/ emotional disorder	3	100%	3	140	5	33%	1	43	0	0.667	32.334	−1.667
Personality disorder	3	67%	2	65	5	67%	2	10	3	0	18.334	−0.667
Schizophrenia	133	83%	181	8,766	291	35%	66	3,206	124	0.864^***^	41.805^***^	−1.256^***^
**Additional psychiatric diagnoses**												
None	87	82%	114	5,295	146	29%	36	1,818	50	0.897^***^	39.966^***^	−1.103^***^
1 extra	48	90%	68	3,343	120	40%	31	1,304	72	0.771^**^	42.479^**^	−1.000^*^
2 or more	23	87%	30	1,519	75	48%	16	450	53	0.609^*^	46.478^**^	−0.957
**Additional medical diagnoses**												
None	87	86%	112	5,293	185	41%	58	2,259	101	0.621^***^	34.874^***^	−0.966^*^
1 extra	40	90%	60	2,643	102	30%	16	640	57	1.100^***^	50.075^***^	−1.125
2 or more	31	74%	40	2,221	54	23%	9	673	17	1.000^**^	49.935^*^	−1.194^*^
**Involuntary admissions to the rehabilitation unit**												
Yes	49	88%	62	3,411	99	31%	24	1,323	52	0.776^***^	42.612^**^	−0.959
No	109	83%	150	6,746	242	37%	59	2,249	123	0.835^***^	41.257^***^	−1.092^**^
**Gender**												
Male	105	88%	151	6,989	248	33%	53	2,223	126	0.933^***^	45.390^***^	−1.162^**^
Female	53	79%	61	3,168	93	38%	30	1,349	49	0.585^***^	34.321^**^	−0.830^*^
**Total**	**158**	**85%**	**212**	**10,157**	**341**	**35%**	**83**	**3,572**	**175**	**0.8164** ^***^	**41.672** ^***^	–1.0506^***^

^*^Significant difference, *p* < 0.05.

^**^Significant difference, *p* < 0.001.

^***^Significant difference, *p* < 0.0001.

The median and interquartile range (IQR) for admission rate during pre- and post-rehabilitation was 1 (0) and 0 (1); for LOS 56 (54.75) and 0 (25.75); and for ER visits 1 (2) and 0 (1), respectively (Table 4).

**Table 4:** The median and IQR of the LOS, admission rate, and ER visits in the pre- and post-rehabilitation year.

	**Pre-rehabilitation**		**Post-rehabilitation**	
**Variables**	**Median**	**IQR**	**Median**	**IQR**
LOS	56	54.75	0	20.75
Admission rate	1	0	0	1
ER Visits	1	2	0	1

Patients with diagnoses of personality disorders, emotional/behavioral disorders, bipolar, and adjustment disorder showed no significant changes in these domains (admission rate, LOS, and ER visits) during the post-rehabilitation year. However, in general, patients with other psychiatric and medical co-morbidities (Tables 3, 5) had lower admission rates and fewer days spent in acute care in the post-rehabilitation year.

**Table 5:** Non-psychiatric co-morbidities.

**Additional medical diagnoses on rehabilitation discharge**	**No discharges**	**% total *N* = 158**
Endocrine, nutritional and metabolic diseases	34	22%
Diseases of the circulatory system	21	13%
Diseases of the nervous system	14	9%
Diseases of the digestive system	12	8%
Diseases of the musculoskeletal system and connective tissue	8	5%
Diseases of the genitourinary system	7	4%
Diseases of the eye and adnexa	6	4%
Certain infectious and parasitic diseases	6	4%
Diseases of the skin and subcutaneous tissue	5	3%
Diseases of the respiratory system	5	3%
Diseases of the ear and mastoid process	3	2%
Diseases of the blood and blood-forming organs and certain disorders involving the immune mechanism	2	1%
Neoplasms	1	1%
**Total discharges with additional medical diagnoses**	**71**	**45%**

In total, there was a significant reduction in the rate of admission and LOS in the acute psychiatric services in the post-rehabilitation year (Tables 3, 4). The total number of acute admissions was reduced from 212 to 83 in the post-rehabilitation year with a mean difference of 0.8 *p* < 0.0001. The net change in the admission rate was reduced in 64% of patients, showed no change in 28%, and increased in 8% of the studied cohort.

Notably, the total number of days spent on acute services plummeted significantly by 6,585 days in the post-rehabilitation treatment year; the mean difference was 41.7, *p* < 0.0001 (Table 3).

Similarly, ER visits for mental health-related problems were significantly reduced in the post-rehabilitation treatment year, with a net change of 166 fewer ER visits, and a mean difference of 1.1, *p* < 0.0001.

**Figure 1:**
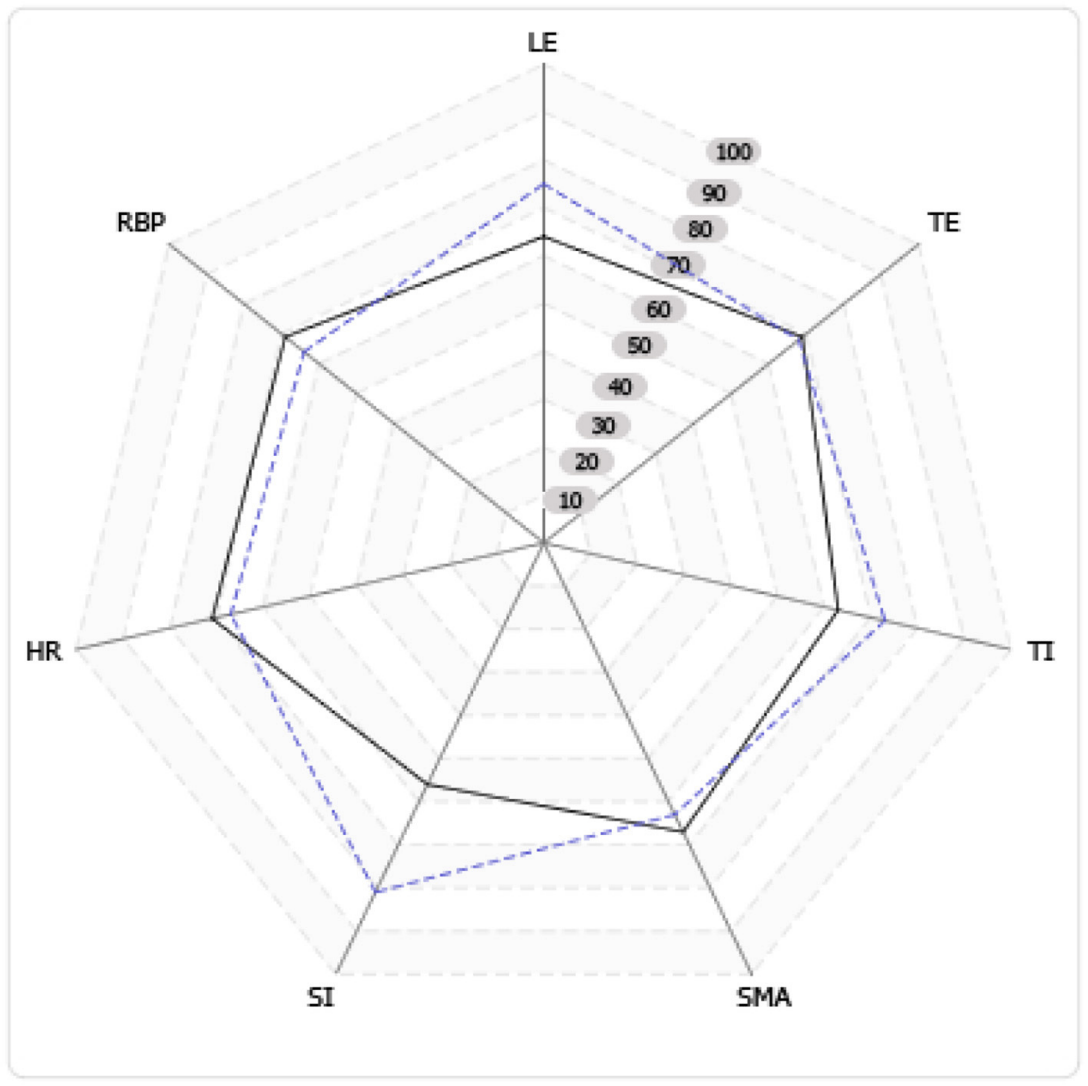
Domain performance in the rehabilitation unit against average in Canada (dotted line represents the studied unit). With reference to Table 6.

The quality of service provision as per QuIRC assessment showed areas of strength that were well-above the national average in living environment, treatment, interventions, and social interface. However, there were areas slightly below average in the therapeutic environment, self-management and autonomy, human rights, and recovery-based practice (Figure 1; Table 6).

**Table 6:** Measurement of quality performance on different rehabilitation domains compared to the national average.

**Key**	**Domain**	**In this unit score (%)**	**Average score in similar Canadian units (%)**
LE	Living environment	75	64
TE	Therapeutic environment	68	69
TI	Treatments & interventions	73	63
SMA	Self-management & autonomy	63	67
SI	Social interface	81	56
HR	Human rights	67	71
RBP	Recovery based practice	64	69

The significant reduction in readmission rate, days spent in hospital, and ER visits were arguably cost-effective and resulted in CAD 9,270,000 in savings in the post-rehabilitation year (Table 7).

**Table 7:** Estimated cost savings.

**Change in a measure in 12 months pre/post-rehabilitation**	***N* discharges**	**% total**	**Net change (post-pre)**
**Inpatient Acute patient days**			–**6,585**
No change	44	28%	0
Decreased	101	64%	−7,156
Increased	13	8%	571
**ER visits**			–**166**
No change	36	23%	0
Decreased	98	62%	−244
Increased	24	15%	78

The average cost per inpatient day billed to MSI, in the amount of CAD 1,400 per patient day, the overall estimated amount saved for all acute inpatient patient days are as follows:

### Inpatient MH acute patient days

Net cost per total MH inpatient acute inpatient days = CAD 1,400 × 6,585 inpatient days = CAD 9,219,000.

The median LOS in the rehabilitation unit was 93.5 with a mean of 165. The estimated cost of the rehabilitation unit was 93.5 X CAD 1,400 = CAD 130,900.

### ER visits

Using the estimated average cost per ER visit billed to MSI, in the amount of CAD 309 per visit, the overall estimated amount saved per ER visit is as follows:

Net cost per total MH inpatient acute patient days = CAD 309 × 166 ER visits = CAD 51,294.

## Discussion

In this 3-year study, an inpatient psychiatric rehabilitation service in Nova Scotia, Canada, resulted in the successful discharge of most patients with severe and persistent mental illness to more socially inclusive environments. It also reduced their post-rehabilitation mental health service utilization, hence greatly enhancing the effectiveness and efficiency of these services.

The deinstitutionalization of patients with severe and persistent mental illness (SPMI) from asylum models of care has resulted in several major challenges for mental health service delivery. Following the deinstitutionalization, it was difficult to find cost-effective and patient-centered alternatives for patients with chronic and disabling mental illnesses. Many patients were discharged to community settings without the necessary support to sustain recovery in the community (3).

Without a well-developed and cost-effective alternative to the asylum model, there was a gap in appropriate services for patients with chronic and disabling mental illness which left some vulnerable patients too functionally impaired to sustain recovery in the community.

Enabling recovery and reducing revolving door admissions of patients with SPMI using the whole-system approach of the biopsychosocial model may not only resonate positively by reducing expenditure but also by improving the quality of life of this group of complex needs patients. Although the cost-effectiveness argument for psychosocial psychiatric rehabilitation may be appealing to stakeholders, as a society we should also value other outcomes, such as social integration/inclusion and quality of life.

A sizable number of the cohort in this study was men with chronic schizophrenia which was consistent with similar studies that examined the demography of inpatient rehabilitation patients (17).

In this study, most patients were discharged into the community, and a large number had no readmissions in the first post-rehabilitation year. There was a significant reduction in readmission rate, LOS, and ER presentation.

In a previous study (2), a sub-cohort of 58 patients from this cohort group were examined for discharge destinations, with one-third of them discharged to their own independent apartments with community support.

A similar study examining 5-year outcomes for mental health rehabilitation service users found that in a 140-patient cohort, 41% were discharged, not readmitted, and moved to less supported placements; 9% achieved independent living; 26% remained stable in the community; and 33% had poor outcomes (i.e., not discharged, readmitted, or lost placement) (18).

Patients with diagnoses of personality disorders, emotional/behavioral disorders, and adjustment disorders showed no significant changes in these domains in the post-rehabilitation year; although the number of patients in each of these groups was relatively low, their inclusion could, nevertheless, confound the results.

The estimated cost savings in inpatient expenditures in the post-rehabilitation year were ~CAD 9,270,000 equivalent to $58,670 saving per patient. Similarly, a retrospective study evaluating the clinical outcome and cost implications for 22 inpatient psychiatric rehabilitation service users in the UK found a significant reduction in bed cost 2 years after rehabilitation compared to the pre-rehabilitation period (19). Furthermore, Bunyan found that a substantial proportion of those patients went into independent living with no further admissions; the total cost saving was 938,000 Sterling (1,601,395, CAD) per year equivalent to $72,790 saving per patient (19) (these figures are based on currency conversion from a website accessed in January 2022).^1^

It is important to realize that the reduction of acute services utilization in the post-rehabilitation year might have a positive impact on patients' and carers' live that goes beyond monetary calculations.

Similar to this study, a naturalistic prospective study examined clinical outcomes and costs for patients with SPMI across 50 rehabilitation service units in England in which a total of 329 patients were followed over 12 months (94% of those recruited). Although service quality was not associated with patients' social function or length of admission at 12 months, successful discharge of over half of complex-needs-patients was achieved within 18 months with an associated reduction in the cost of care. Factors associated with successful discharge were the recovery orientation of the service and patients' activity and social skills at recruitment (20).

The Royal College of Psychiatrists' Center for Quality Improvement (CCQI) runs accreditation networks for inpatient mental health services that include rehabilitation services. The Accreditation of Inpatient Mental Health Services (AIMS) program has incorporated QuIRC as one of its standardized measures for Mental Health services to demonstrate that they meet national guidelines and standards for quality of care (21).

In this study, QuIRC (7–11) was employed to measure the quality of care and the service identified areas of strength to build upon, including the living environment, treatment intervention, therapeutic environment, and social interface. The service also intuitively recognized its limitations in areas such as self-management, human rights, and recovery-based practice that require more endeavors for improvement; however, the level of care provided by this unit was above the average of national quality on the social interface, living environment, and treatments/interventions as per the QuIRC assessment.

Notably, the deinstitutionalization movement across the world has been followed by trans-institutionalization, i.e., patients with SPMI end up in different forms of institutions rather than living in their own homes, such as nursing homes, boarding houses, and homes for the elderly or even in prisons (22).

In similar studies (2, 18–20), psychiatric inpatient rehabilitation services demonstrate that they can successfully bridge the gap for patients with chronic and severe mental illness and help enable recovery and reintegration to less restrictive and more independent places of residence using the whole-system approach of the biopsychosocial model. This was associated with a remarkable reduction in revolving door readmissions and LOS.

In this study, patients served as self-control during the pre- and post-rehabilitation period and this design would reduce possible biases that may result from comparing the heterogeneous group of patients. Hence, direct causality of the effectiveness of psychiatry rehabilitation intervention on the reported outcomes could be inferred when demonstrating the difference in service utilization in the pre- and post-rehabilitation. These findings were also in keeping with Dalton-Locke's systematic review in 2021.

Limitations of the study could be the relatively small sample size and the presence of a high prevalence of other psychiatric and medical co-morbidities in the studied cohort; hence, the heterogeneity of the sample may impact the outcome. The non-randomized nature of the study design and the lack of a control group are also considered limitations.

However, these limitations may also reflect the true nature of patients with complex co-morbidities and the ethical dilemma that one may face when randomizing patients who need rehabilitation treatment into “a treatment as usual arm”. Given the illness severity and complexity of patients with psychiatric rehabilitation needs, randomization of such patients to rehabilitation and non-rehabilitation services would be impractical and ethically questionable.

Despite these limitations, our data and the findings of other studies indicate that inpatient psychiatric rehabilitation services are an effective and long-term investment in the mental healthcare of patients with complex psychosis.

Further prospective studies on larger patient cohorts are recommended to establish the longer-term impact of psychiatric inpatient rehabilitation services over several years. Future research could also include other measures important to the concept of recoveries such as social participation and measures of quality of life in the community. Further studies are needed to elucidate which components of psychiatric rehabilitation most optimally support a recovery-oriented approach.

## Data availability statement

The original contributions presented in the study are included in the article/supplementary material, further inquiries can be directed to the corresponding author.

## Ethics statement

The studies involving human participants were reviewed and approved by Research Ethics Board of Nova Scotia Review Exemption Approval Letter REB FILE #: 1028070. Consent to participate and to publish was not applicable as patients' information is anonymized and Research Ethics Board approved exemption for publication. Written informed consent for participation was not required for this study in accordance with the national legislation and the institutional requirements.

## Author contributions

The first draft of the manuscript was written by MA who along with LD, DE, NL, and JG contributed to the study conception and design, material preparation, data collection, and analysis. All authors commented on previous versions of the manuscript, and they all read and approved the final manuscript.

## Footnotes

^1^Currency Converter. Available online at: https://www.nice.org.uk/guidance/cg178.
